# Quality indicators for appropriate antibiotic prescribing in urinary tract infections in children

**DOI:** 10.1186/s12879-023-08356-z

**Published:** 2023-06-12

**Authors:** Konstantinos Vazouras, Charlotte Jackson, Laura Folgori, Anastasia Anastasiou-Katsiardani, Yingfen Hsia, Romain Basmaci

**Affiliations:** 1 Children’s Haematopoietic Stem Cell Transplant Unit, Great North Children’s Hospital, Newcastle Upon Tyne Hospital NHS Foundation Trust, Newcastle Upon Tyne, UK; 2 Paediatric Infectious Diseases Research Group, Institute of Infection and Immunity, St George’s University of London, Cranmer Terrace, London, SW17 0QT England; 3 Paediatric Infectious Disease Unit, Department of Paediatrics, Luigi Sacco Hospital, University of Milan, Milan, Italy; 4 Paediatrics Department, Achillopouleion General Hospital of Volos, Volos, Greece; 5 Queen’s University Belfast, School of Pharmacy, 97 Lisburn Road, Belfast, BT9 7BL UK; 6 Service de Pédiatrie-Urgences, Hôpital Louis-Mourier, Assistance Publique – Hôpitaux de Paris, 92700 Colombes, France; 7 Université de Paris, Infection, Antimicrobiens, Modélisation, Evolution, Unité Mixte de Recherche 1137, Institut National de La Santé Et de La Recherche Médicale, 75006 Paris, France

**Keywords:** Antibiotics, Urinary tract infections, Children, Appropriate prescribing, Quality indicators, Antimicrobial stewardship

## Abstract

**Background:**

The aim of this study was to define a set of urinary tract infections (UTIs)-specific quality indicators for appropriate prescribing in children and evaluate clinical practices in a district general hospital in Greece.

**Methods:**

The UTIs-specific quality indicators were informed by a review of the existing literature. Quality indicators were selected to describe the overall antibiotics use, prescribing patterns and UTIs clinical management regarding treatment and prophylaxis in a cohort of children admitted with a UTI. Microbiological, clinical and prescribing data about dosing, duration and route of administration were collected from the patients’ electronic health records.

**Results:**

Twelve quality indicators were adapted or developed for prescribing in childhood UTIs. A broad variety of antibiotics were prescribed for UTIs, with a drug utilization (DU) 90% rate of 6 and 9 different antibiotics for febrile and afebrile UTIs, respectively. Despite the low incidence of multi-drug resistant UTIs in the study period (9/261, 3.4%), broad-spectrum antibiotics were prescribed in 33.5% (164/490) of prescriptions. A total of 62.8% (164/261) of patients were started on empiric combined therapies, while opportunities to de-escalate were missed in 37.8% (62/164) of them. One quarter (67/261, 25.7%) of patients did not fulfil the criteria for receiving treatment, while nearly half of those prescribed prophylaxis (82/175, 46.9%) could have avoided having a prophylaxis prescription.

**Conclusions:**

Our study identified substantial gaps for improvement in antimicrobial prescribing for UTIs in children. The application of the proposed quality indicators could help to limit unnecessary antibiotics use in children with UTI.

## Background

Urinary tract infection (UTI) is common in childhood, affecting either the upper (defined as pyelonephritis) or lower urinary tract (defined as cystitis) [1]. UTIs have been estimated to account for 5–14% of pediatric emergency department visits annually [2], necessitating the prescription of large volumes of antibiotics in healthcare [3, 4]. However, large-scale prescription of antibiotics is strongly associated with the occurrence of antimicrobial resistance (AMR) in bacteria [5], often leading to an increase of resistance to first line antibiotics for UTIs [6] such as trimethoprim and penicillins [7]. Overprescription is also accountable for the emergence of resistance to cephalosporins, such as the extended-spectrum β-lactamase (ESBL)-producing bacteria [8, 9]. The latter are being increasingly isolated from urine specimens in children with UTIs [10], thus pointing to the need to increase control over antimicrobial prescribing for UTIs.

The World Health Organization (WHO) has advocated the need for healthcare providers to strengthen surveillance and research on antibiotics use [11]. The development and monitoring of quality indicators (QIs) could help improve the quality of care provided [12, 13] and reduce AMR by measuring and reducing inappropriate prescribing [14]. QIs for appropriate prescribing have been suggested for cystitis [15] and acute uncomplicated or complicated pyelonephritis in adults [16]. The Worldwide Antibiotic Resistance and Prescribing in European Children (ARPEC) network have developed QIs to measure appropriate inpatient antibiotic use in neonates and children with a bacterial infection [4]. Nevertheless, no disease-specific QIs have ever been suggested for children, limiting our potential to control antibiotic prescribing for each infection individually.

The aims of this study were to: a) adapt existing QIs and develop new UTIs-specific QIs for appropriate prescribing in children, and b) apply those indicators to measure appropriateness of care in a hospital setting.

## Methods

### Study setting and design

This was a retrospective observational cohort study conducted in a general district hospital in Central Greece, the Achillopouleion General Hospital of Volos (AGHV). AGHV is a 400-bed (27 paediatric beds/ 11 neonatal cots) hospital, admitting 24,000 patients per year [17]. The present study included inpatients from 1 month to 18 years old admitted with an ICD-10 diagnosis of UTI between August 2010 and September 2016. The patients’ notes were retrieved from the Paediatric Department’s Electronic Clinical Archive, which has also been used in a previously published study [18]. Patients were excluded from analysis if: a) they were neonates (up to 28 days old), b) immunocompromised patients, c) they had a concurrent proven bacterial infection prompting additional antibiotic treatment, or d) treatment data was missing. Information on susceptibility patterns, prescriptions, duration, dosing, route of administration and indication for prescribing (empiric or targeted treatment, treatment or prophylaxis) was collected.

### Development of QIs and definitions

The UTIs-specific QIs were selected and informed by the review of the existing literature on children and adults. A literature search was performed in PubMed using the following terms: “quality indicators”, “urinary tract infections”, “appropriate prescribing”, and “children”. Results of the literature search informed the choice of indicators as described below. The newly derived indicators were developed based on the descriptive analysis of the present sample. The QIs were categorised according to: a) the antibiotics used and their critical importance (defined below), b) prescribing patterns relating to duration, dosing and combination of different antibiotics, c) need for treatment and de-escalation decisions, d) need for prophylaxis and selection of agent (Table 1).

**Table 1:** UTIs-specific quality indicators for appropriate prescribing in children

**Antibiotics use**	
DU75%, DU90% [4]	Antibiotics accounting for 75% and 90% of prescriptions for a UTI
DOT per patient [19, 20]	The number of days that a patient receives antibiotics (“antibiotic days”)
Percentage of patients receiving prescriptions in each AWaRe category [21]	Patients prescribed “Access” antibiotics × 100/ Total number of patients
	Patients prescribed “Watch” antibiotics × 100/ Total number of patients
	Patients prescribed “Reserve” antibiotics × 100/ Total number of patients
Use of second-line antibiotics for 3GC resistant UTIs^b ^[22]	Prescriptions of second-line^a^antibiotics × 100/ Total number of prescriptions
**Antibiotic prescribing patterns**	
Percentage of patients on antibiotic combination therapies^b ^[4]	Number of patients treated with combined therapy × 100/ Total number of patients
Percentage of patients receiving IV treatment for less than or equal to 3 days^c^	Number of patients treated intravenously for less than or equal to 3 days × 100/ Total number of patients
Percentage of prescriptions out of the recommended dosing range^c^	Intravenous prescriptions out of the recommended dosing range × 100/ Total number of prescriptions
Percentage of prescriptions with dosage adjustments to renal function [16]	Number of prescriptions with dosing adjustments × 100/ Total number of prescriptions for which dosage adjustment was required
**Clinical management (treatment)**	
Percentage of patients treated for a UTI who did not meet the diagnostic criteria^c^	Number of treated patients not fitting the diagnostic criteria × 100/ Total number of patients treated for a UTI
Percentage of patients for whom treatment was de-escalated [23]	Number of patients to whom combined therapy was de-escalated × 100/ Total number of patients with combined therapies
**Clinical management (prophylaxis)**	
Percentage of patients prescribed prophylaxis without a clinical indication^c^	Number of patients having an unnecessary prescription for prophylaxis × 100/ Total number of children with a prophylaxis prescription
Percentage of patients receiving prophylaxis for whom an appropriate drug was selected^c^	Number of patients with appropriate prophylaxis regimen × 100/ Total number of patients with a prophylaxis prescription

*Abbreviations*: *UTI* Urinary tract infection, *DU* Drug utilization, *DOT* Days of therapy, *3GC* Third-generation cephalosporin, *VCUG* Voiding cystourethrogram

^a^second line-antibiotics: aztreonam, carbapenems, piperacillin-tazobactam, amikacin, ciprofloxacin, cefepime, fosfomycin, tigecyclin, colistin, ceftazidime-avibactam, pivmecillinam and temocillin

^b^modified from existing literature

^c^newly developed

For this study, “prescription” was defined as the use of one substance in one route of administration (4), while “combined therapy” was defined as the concurrent use of more than one antibiotic for the treatment of one patient [24]. Prophylaxis was defined as the continuous, low-dose daily administration of antimicrobials for long periods of time [25]. Third-generation cephalosporin (3GC) resistant UTI was defined as any UTI caused by a pathogen non-susceptible to ceftriaxone or cefotaxime [26].

### Antibiotics use

A panel of experts recently suggested that antibiotic use should be expressed in at least two metrics simultaneously [27]. Firstly, to assess antibiotics use, we ranked the number of antibiotics used for UTIs, accounting for 90% and 75% of (antibiotic) drug utilization (DU90% and DU75%, respectively) [4]. In addition, the days of therapy (DOT) were calculated per patient to describe antibiotics use. When a patient received more than one antibiotic, the sum of the “antibiotic days” was counted for this patient [19]. Among the antibiotics used, we defined some second-line antibiotics for the treatment of 3GC resistant UTIs, including the following: aztreonam, carbapenems, piperacillin-tazobactam [22, 28], amikacin [28, 29], ciprofloxacin, cefepime, fosfomycin, tigecyclin, colistin [28], ceftazidime-avibactam [30], pivmecillinam [30] and temocillin [31]. Antibiotic consumption was further analysed according to WHO’s Access, Watch and Reserve (“AWaRe”) groups [31]. The AWaRe classification aims to promote antimicrobial stewardship by encouraging use (where needed) of appropriate, often narrow-spectrum, antibiotics, and limiting use of other antibiotics [31].

### Antibiotic prescribing patterns

We assessed the prevalence of combined antibiotic therapies, considered to be an indicator of low-quality of prescribing [4], especially given that current national and international clinical management guidelines predominantly recommend monotherapy for the empiric treatment of UTIs [32–34]. For hospitalised children with a non-bacteraemic UTI, intravenous antibiotic courses of up to 3 days appear adequate, with no benefit from longer courses (e.g. 10 days) [32]. Hence, we considered it a marker of good quality prescribing if children received intravenous antibiotics for no longer than 3 days. Children with either clinically indicated or proven bacteraemia were excluded from the intravenous duration analysis, as intravenous courses for bacteraemic UTIs may vary, lasting up to 7-to-10 days [35]. Finally, appropriateness of dosing in each prescription, measured in milligrams per kilogram per day, was assessed according to the Greek National Organisation for Medicines (GNF) [36]. For antibiotics where no ranges for administration were specified in the guidance, we used the WHO recommendations for children’s dosing or the drug’s summary of product content [37]. For antibiotics (i.e. ampicillin-sulbactam) for which no relevant dosing ranges could be found, we selected a divergence of 10% from the proposed dosing to be acceptable [38]. The adaptation of the dosage on the basis of renal function was also considered a good marker to measure dosing appropriateness [16].

### Clinical management (treatment and prophylaxis)

Patients were classified as needing treatment if they had positive clinical and/or microbiological features suggesting a febrile or afebrile UTI [18]. Among the children who were initially treated empirically with a combined therapy, we further counted the percentage of patients for whom therapy was de-escalated therapy once the antibiogram was available [23]. De-escalation from a broad to a narrow-spectrum antibiotic [39] could not be calculated due to paucity of narrow therapeutic options (eg benzylpenicillin, penicillin V or nitrofurantoin) in this particular setting.

The need for prescription of prophylaxis was assessed according to age. For children under 3 years old, this was based on the occurrence of an atypical or recurrent UTI as suggested by the National Institute for Health and Care Excellence (NICE) [33]. Children aged < 3 years old with an episode of UTI could also be considered eligible for prophylaxis if they fulfilled at least one of the following criteria: a) known presence of active vesicoureteral reflux (VUR) or major structural urinary tract abnormality, b) family history of VUR [25], c) abnormal renal ultrasound with findings suggestive of potential underlying VUR [40], d) kidney transplant, or e) young infants with prenatal hydronephrosis awaiting for voiding cystourethrogram [41]. Children older than 3 years old were considered to need prophylaxis if they had major structural urinary tract abnormality (e.g. dysplastic kidneys, single kidney, combined anomalies of the urinary tract) or kidney transplant [42].

If prophylaxis was indicated, trimethoprim, trimethoprim/sulfamethoxazole or nitrofurantoin were considered the preferred choices [42, 43], as beta-lactams, and especially cephalosporins, have been associated with the recurrence of UTIs due to extended-spectrum beta-lactamase-producing bacteria or multi-drug resistant (MDR) uropathogens other than *Escherichia coli* [33].

## Results

**Fig. 1:**
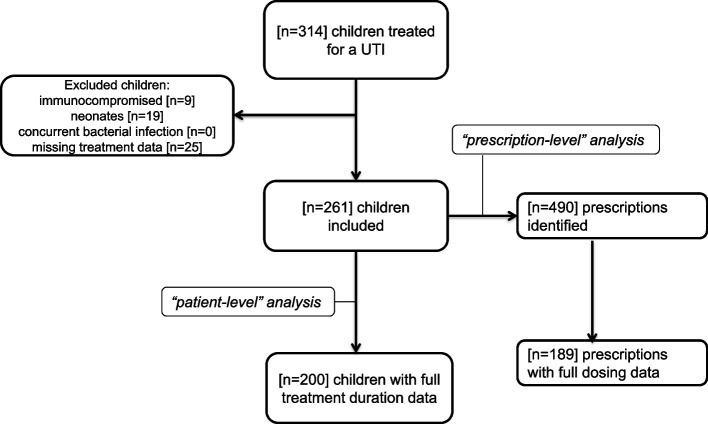
Formation of the cohort

Among 314 patients treated for a UTI, 261 (83.1%) fulfilled the criteria for inclusion (Fig. 1). The full age, sex distribution and background of the patients are shown in Table 2. Four-hundred ninety prescriptions were identified for the treatment of a UTI in these patients. Amikacin was the top prescribed antibiotic, while the detailed antibiotic’s use (Drug Utilization 75% and 90%) is described in Table 3. A median of 9 DOTs was calculated per patient (IQR 6.0 -11.5). Among the total hospital prescriptions, 164/490 (33.5%) were related to second-line antibiotics for the treatment of 3GC resistant UTIs, mostly amikacin (155/490, 31.6%). Notably, only 9/261 (3.4%) patients had an infection with a UTI resistant to 3^rd^ generation cephalosporins. No carbapenem-resistant strains were identified.

**Table 2:** Demographic and clinical features of children with UTIs included in the analysis

Demographics	Febrile UTIs (*n* = 198)	Afebrile UTIs (*n* = 63)	All UTIs (*n* = 261)
	**% (*n*)**	**% (*n*)**	**% (*n*)**
**Age (on admission)**			
1 month – 2 years	67.2 (133)	60.3 (38)	65.5 (171)
2 years – 5 years	16.1 (32)	19.1 (12)	16.9 (44)
> 5 years	16.7 (33)	20.6 (13)	17.6 (46)
**Sex**			
Male	24.2 (48)	38.1 (24)	27.6 (72)
Female	75.8 (150)	61.9 (39)	72.4 (189)
**Background**			
Children with no underlying condition or comorbidity	79.8 (158)	79.4 (50)	79.7 (208)
Structural UT-abnormalities^a,c^	12.1 (24)	4.8 (3)	10.3 (27)
Other medical conditions^b,c^	8.6 (17)	15.9 (10)	10.3 (27)
Concurrent infections	5.1 (10)	3.2 (2)	4.6 (12)
Bacteraemic UTIs	2.5 (5)	0.0 (0)	1.9 (5)
Resistant UTIs^d^	4.0 (8)	1.6 (1)	3.4 (9)
Recurrent UTIs	19.7 (39)	11.1 (7)	17.6 (46)
Atypical UTIs	39.9 (79)	30.2 (19)	37.6 (98)
Recent hospitalisation	4.5 (9)	4.8 (3)	4.6 (12)
Recent antibiotics use^e^ or concurrent prophylaxis^f^	16.7 (33)	7.9 (5)	14.6 (38)

^a^UT-abnormalities: vesicoureteral reflux, major anatomic UT-abnormalities

^b^Other concurrent transient or chronic conditions: gastrointestinal diseases, heart defect, endocrinology disorders, syndromes, haematological conditions, prematurity

^c^Children could have more than one co-morbidity

^d^Due to pathogens resistant to 3^rd^ generation cephalosporins or carbapenems

^e^within 2 months

^f^receiving already prophylaxis on presentation

**Table 3:** Antibiotic prescriptions for UTIs, ranked at overall drug utilization 75% and 90% (DU75% and DU90%)

Febrile UTIs (*n* = 384)	Afebrile UTIs (*n* = 106)	UTIs (*n* = 490)
1. Amikacin 33.3% (128)	1. Amikacin 25.5% (27)	1. Amikacin 31.6% (155)
2. Amoxicillin/clavulanic acid 17.7% (68)	2. Amoxicillin/clavulanic acid 20.8% (22)	2. Amoxicillin/clavulanic acid 18.4% (90)
3. Ampicillin 11.5% (44)	3. Cefuroxime 16.0% (17)	3. Ampicillin 10.8% (53)
4. Cefotaxime 10.4% (40) 5. Ampicillin/sulbactam 9.4% (36)	4. Ampicillin 8.5% (9) 5. Ampicillin/sulbactam 8.5% (9)	4. Cefuroxime 10.0% (49) 5. Ampicillin/sulbactam 9.2% (45)
**Total 82.3%**	**Total 79.3%**	**Total 80.0%**
6. Cefuroxime 8.3% (32)	7. Cefotaxime 3.8% (4)	6. Cefotaxime 9.0% (44)
	8. Cefprozil 3.8% (4) 9. Piperacillin-tazobactam 2.8% (3)	
**Total 90.6%**	**Total 89.7%**	**Total 89.0%**

*Abbreviations*: *UTIs* Urinary tract infections

Two-hundred nine (80.1%) patients were prescribed at least one “Access” antibiotic, approximately 50% of patients (132/261) received at least one “Watch”. None of them had any “Reserve” prescription. One hundred sixty-four (62.8%) patients were started empirically on combined antibiotics, while 5/261 (1.9%) had initially targeted combined therapies. Amongst the patients initially receiving empiric combined therapy, treatment was de-escalated in 102/164 (62.2%) patients. Among 200 children with a UTI for whom the exact duration of antibiotics was available, only 28/200 (14.0%) had intravenous antibiotics for shorter than 3 days. The median duration of intravenous antibiotics was 6.0 days (IQR: 4.0–7.0) for these children. Dosing information could be retrieved for 189/490 prescriptions (38.6%). Among them, dosing was appropriate in 154/189 (81.5%) prescriptions. The dosing was found lower than the recommended range in 7/189 (3.7%) and higher in 17/189 (9.0%) prescriptions, according to the GNF. Most of the exceeded upper high rates in dosing were observed regarding amoxicillin/clavulanic acid and cefuroxime, as the given ranges appeared narrower in the GNF comparing to other available national guidance [44]. The adaptation of dosage according to renal function could not be measured for this sample, due to scarce data on the patients’ somatometric parameters.

Included patients were also assessed for their need to receive treatment and/or prophylaxis. Sixty-seven children (67/261, 25.7%) did not fulfil the criteria for a UTIs diagnosis, suggesting their potential unnecessary treatment. Among the 175 patients who were prescribed prophylaxis, 82 (82/175, 46.9%) did not have a substantiated indication for it. Trimethoprim/sulfamethoxazole or nitrofurantoin were prescribed in only 21/175 (12.0%) of the patients receiving prophylaxis. One hundred forty-nine (149/175, 85.1%) children had a prescription of a beta-lactam, mostly 2^nd^ generation cephalosporins (126/175, 72.0%).

## Discussion

### Principal findings

We developed a set of 12 indicators to better explore UTIs antimicrobials prescribing in children. These metrics revealed significant areas for improvement in all steps of prescribing: UTI diagnosis, treatment and prophylaxis selection, route of administration, duration and dosing optimisation. Remarkably, although 3GC resistant UTIs was rare in this population (3.4%), a second-line antibiotic or a “Watch” antibiotic was prescribed in 33.5% and 50.6% of patients, respectively. More than half (62.8%) of patients received empiric combined therapies, while the duration of intravenous treatment was overly long in 86.0% of children.

### Strengths and limitations of this study

To the best of our knowledge, this is the first study defining UTIs-specific QIs for appropriate prescribing in children. These indicators are applied in an inpatient’s population in a district hospital, highlighting the deficiencies in antimicrobial prescribing in this setting. The proposed set of indicators appears relevant both for individual prescribers and policy-makers as they give insight both to antibiotics consumption and clinical management. They could potentially be incorporated into paediatric antibiotic stewardship programs (PASPs). The application of these QIs could substantially help to improve clinical practice, reduce costs, antimicrobial exposure and selection of resistance [45].

The main limitation of this study is that the values of the QIs cannot directly be generalised to other settings, as it describes the local prescribing practices in this specific unit. Prescribing practices may vary in different countries or continents [4]. Prescribing practices may also differ in tertiary care hospitals, where children with more complex backgrounds (neonates, immunocompromised, transplanted or oncology patients, intensive care) often receive empiric treatments with more advanced antibiotics or complex regimens to cover MDR strains [22, 23, 46]. Therefore, if the QIs we developed are used to assess the quality of prescribing, patient characteristics should be borne in mind. Another limitation is the study period. Stricter prescribing policies suggested in this study are based on current literature, which suggests monotherapy and shorter courses for UTIs (6,30), while intravenous courses are not warranted for lower UTIs [47]. At the time of the study (2010–2016), national guidance suggested longer courses (10–14 days) and combined therapy for acutely ill children with a UTI [48]. This may have affected clinical practice in this setting, leading to the overprescription of combined therapies. Greek guidance for UTIs treatment has subsequently been revised in 2015 [34]. Finally, data was collected retrospectively, which led to dosing data missing for more than 60% of prescriptions. However, this paper is the first to suggest a standardized way to assess metrics and quality of prescribing for UTIs in children which can be validated in relation to patient outcomes and implemented in wider, multi-centre studies.

### UTIs-specific QIs in children and adults

In 2007, the European Surveillance of Antimicrobial Consumption (ESAC) network published a set of QIs for antibiotic use in adult women with cystitis [15]. This was followed by another set of QIs for adults with uncomplicated and complicated pyelonephritis, developed by a panel of national experts [16]. These sets of indicators are not applicable to the paediatric population due to differences in the natural history of UTIs between children and adults. Evidence suggests that UTIs in adults are associated with risk factors such as sexual intercourse, diabetes mellitus, permanent catherization, immunocompromise and acquired nephropathies [49]. In contrast, the most common risk factors in children are high-grade vesicoureteral reflux [50], infancy, fever [6] and functional abnormalities such as constipation [33]. UTIs in adult men are usually treated as more complicated, as they may result from anatomic abnormalities, while a low threshold for treatment is being kept for pregnant women [51]. Guidelines in diagnosis and treatment also vary in children [6, 33, 52] (Table 4). Βeta-lactams efficacy has been challenged for cystitis in women [53], whilst they appear to be the first choice both for febrile or upper or lower UTIs in children [6, 33]. This may suggest that the development of quality indicators for appropriate prescribing for UTIs in children should target different drugs compared to adults.

**Table 4:** Guidelines on UTIs treatment for children and adults

**Population**	**Localisation**	**NICE (ch)** [1] **/ SIGN (ad)** [2]	**IDSA (ad)** [3] **/ AAP (ch)** [4]	**WHO EML** [5, 6]	**EODY** [7]
**Children**	fUTIs/ uUTIs/ pyelonephritis	cephalosporins, co-amoxiclav, ceftriaxone	parenteral: ceftriaxone, cefotaxime, ceftazidime, gentamicin, tobramycin, piperacillin; or oral: co-amoxiclav, co-trimoxazole, sulfisoxazole, cephalosporins (cefixime, cefpodoxime, cefprozil, cefuroxime axetil, cephalexin)	severe pyelonephritis: cefotaxime, ceftriaxone, amikacin; mild to moderate: ciprofloxacin	Cephalosporin of 2^nd^ or 3^rd^ generation iv followed by oral agents OR oral agents
	aUTIs/LUTIs/ cystitis	trimethoprim, nitrofurantoin, cephalosporin or amoxicillin	-	1^st^ line: amoxicillin, co-amoxiclav, nitrofurantoin, trimethoprim, co-trimoxazole	-
**Adults**	fUTIs/ uUTIs/ pyelonephritis	co-amoxiclav or ciprofloxacin	ciprofloxacin or levofloxacin oral, ceftriaxone, aminoglycosides, co-trimoxazole oral, β-lactams^a^, carbapenems	severe pyelonephritis: cefotaxime, ceftriaxone, amikacin, ciprofloxacin	ciprofloxacin, levofloxacin, beta-lactam + BLI, 3^rd^ generation cephalosporins ± aminoglycosides ± penicillin
	aUTIs/LUTIs/ cystitis	1^st^ line: nitrofurantoin, co-trimoxazole, 2^nd^ line: co-amoxiclav, quinolones and cephalosporins	nitrofurantoin, co-trimoxazole, fosfomycin^a^, pivmecillinam^a^, fluoroquinolones^b^, β-lactams^a^ ( co-amoxiclav, cefdinir, cefaclor, cefpodoxime, cephalexin)	1st line: amoxicillin, co-amoxiclav, nitrofurantoin, trimethoprim, co-trimoxazole	co-amoxiclav, quinolones, nitrofurantoin, co-trimoxazole

*Abbreviations*: *ch* Children, *ad* Adults, *UTIs* Urinary tract infections, *fUTIs* Febrile UTIs, *aUTIs* Afebrile UTIs, *uUTIs* Upper UTIs, *LUTIs* Lower UTIs

^a^may have inferior efficacy

^b^collateral damage

### Clinical implications

The application of QIs in this study identified substantial areas for improvements in prescribing. A considerable number of patients (62.8%) had combined empiric therapy, which was continued after the antibiogram results (37.8%). The empiric treatment of UTIs with combined antibiotics is not routinely suggested by the existing guidelines [6, 33], as it has not proven more effective compared to the use of one single agent [54, 55]. Their only potential utility may be for critically-ill patients at risk for MDR-infections [46]. It is also essential for clinicians not to lose opportunities for de-escalation of treatment when AMR information is available [23].

The use of combined, lengthy antibiotic therapies, where not indicated, represents an extra financial burden for healthcare in Greece [3] and unnecessary burden for patients and carers. Moreover, long antibiotic courses have been associated with the recurrence of resistant bacteria from the same patients [5]. When intravenous antibiotics are given, shorter courses (two to four days) of intravenous therapy followed by oral therapy are as effective as longer courses (seven to 10 days) of intravenous therapy [32, 55–60], in preventing recurrence of bacteriuria and renal damage. Furthermore, 12.7% of patients had inappropriate dosing (lower or higher than the specified ranges) in our study, which is similar to a US study where 11.5% of children with a UTI were ambulated with an inappropriate antibiotic dose [38]. Dosing divergences may be associated either with prescribing errors or the wide variation observed in available paediatric dosing recommendations [61, 62].

Finally, a large number of children had a non-indicated prophylaxis prescription for a UTI (46.9%), mostly a cephalosporin (72.0%). The need for prophylaxis has been challenged in recent literature. Children seem not to benefit from prophylaxis as there is no evidence that it prevents renal scarring [25, 42] or long-term sequelae [63]. When prophylaxis is needed, trimethoprim/sulfamethoxazole or nitrofurantoin are the most appropriate choices, unless contraindicated or the child has already had urinary isolates test positive for resistance to these drugs [42].

### Antimicrobial resistance and global health policies

A broad variety of antibiotics were prescribed for UTIs, with a DU90% ranging from 6 to 9 different antibiotics. A DU75% of 9 to 11 antibiotics has been found in children with infection in the Eastern Mediterranean region [4, 64]. Beta-lactams accounted for nearly half of the prescriptions in those patients [4, 64].

Treatment could have been more uniform in this study because this was a single-centre study and the included patients were immunocompetent. A recent meta-analysis showed that the overall cure rate in children with febrile UTIs [65] was 95.3% regardless of the investigational drug chosen, the route of administration, duration and dosing. The studied clinical trials included mostly penicillins, cephalosporins and aminoglycosides. The wide range of the used antibiotics also reflects the lack of uniformity in the national and global recommendations [6, 33, 48, 51, 53, 66] (Table 4).

Limiting the unnecessary use of broad-spectrum antibiotics in patients with unremarkable background should be a core target in UTIs antimicrobial stewardship. Although 3GC resistant UTIs accounted for 3.4% and bacteraemic UTIs for 1.9% of UTIs in the study period, a second-line antibiotic was prescribed in 33.5% of prescriptions. The high rates of amikacin prescribing seem disproportionate with the severity of disease in the included population. Amikacin should be reserved for severe pyelonephritis [52] or resistant UTIs [67–69] in children. No other second-line antibiotics, such as fosfomycin or tigecycline, were prescribed in this population. Oral fosfomycin has been recommended as UTIs first-line treatment in adult women [51]. However, the WHO advocates that these antibiotics should be reserved as “last resort” for threatening infections when all alternatives have failed. The widespread use of “Watch” or “Reserve” antibiotics in a population signifies low-quality of prescribing, due to their high resistance potential or critical importance for public health, respectively [31, 52, 70].

## Next steps and future research

Further studies need to be done to develop QIs for antimicrobial prescribing in children. These QIs need to be specific for each infectious syndrome and tailored to fit the clinical challenges of each one of them as well as cost-effective and efficient. The standardisation of every step of clinical practice and the development of benchmarks for optimal prescribing on infections are of paramount importance to limit AMR in the paediatric population. The suggested QIs need to be flexible and relevant both for individual prescribers and policy makers. Their implementation should be further validated in wider, multi-centre studies in different settings, countries and continents.

## Data Availability

The data analysed/generated in this study is available from the corresponding author upon request.
